# COP9-Signalosome deneddylase activity is enhanced by simultaneous neddylation: insights into the regulation of an enzymatic protein complex

**DOI:** 10.1186/s13008-015-0011-0

**Published:** 2015-08-11

**Authors:** Gil Bornstein, Chagai Grossman

**Affiliations:** The Talpiot Medical Leadership Program, Department of Internal Medicine D, The Chaim Sheba Medical Center, Tel-Hashomer, affiliated with the Sackler Faculty of Medicine, Tel-Aviv University, Tel Hashomer, 52621 Israel; The Rheumatology Unit, The Chaim Sheba Medical Center, Tel-Hashomer, affiliated with the Sackler Faculty of Medicine, Tel-Aviv University, Tel Hashomer, Israel

**Keywords:** NEDD8, Deneddylation, Signalosome, Cul1, ATP

## Abstract

**Background:**

Cullin-RING ubiquitin ligases (CRLs) are regulated by neddylation, which is a post translation modification of the Cullin family proteins. Neddylation of Cul1 activates the ligase through some means of biochemical mechanisms. The rate of neddylation and its extent are regulated by 2 opposing enzymatic processes: neddylation by an enzymatic cascade, and deneddylation by COP9-Signalosome (CSN) complex protein. The mechanism by which COP9-Signalosome catalytic activity is regulated is not well understood.

**Methods:**

We set an in vitro neddylation and deneddylation reaction using as a source for specific COP9/Signalosome deneddylase activity either Hela cells extract or purified Signalosome. Neddylation reaction of either endogenic Cul1 from Hela cells extract or recombinant Cul1 was catalyzed by recombinant neddylation enzymes. Deneddylation rate was tested either simultaneous to neddylation or after termination of neddylation by using an ATP depleting reaction or by directly inhibiting the neddylation activation enzyme named APP-BP1/UBA3 by its specific inhibitor MLN-4924.

**Results:**

We demonstrated that neddylation and deneddylation are catalytically engaged and that inhibition of Cul1 neddylation significantly causes a decline in the rate of COP9-Signalosome deneddylase activity. Since neddylation is an ATP consuming reaction we managed to isolate the 2 opposing processes which surprisingly caused a decline in COP9 activity. Using MLN-4924 we demonstrated that direct inhibition of neddylation negatively influences the rate of deneddylation. The hypothesis that phosphorylation controls deneddylation was ruled out by the fact that no change in the rate of deneddylation was exemplified while converting the use of ATP with AMP-PNP.

**Conclusions:**

We demonstrated that deneddylation of Cul1 is positively regulated through direct simultaneous neddylation and is not dependent upon autophosphorylation. Defining the mechanism that regulates neddylation and deneddylation of Cullin proteins is important due to their effect on highly conserved cellular processes. We showed that minor changes in the degree of Cul1 neddylation linearly control the degree of p27 conjugation to ubiquitin, which emphasizes the hypothetic physiologic significance of our findings.

**Electronic supplementary material:**

The online version of this article (doi:10.1186/s13008-015-0011-0) contains supplementary material, which is available to authorized users.

## Background

Cullin-RING ubiquitin ligases (CRLs) comprise the largest family of ubiquitin protein ligases. Among them, the SCF (Skp1-Cullin1-F-box protein) class is the best studied [1, 2]. Cullin1 (Cul1) serves as a scaffold protein. Its N-terminal domain binds the Skp1 adaptor protein and its C-terminal domain binds the ROC1 RING finger protein. Specificity of target proteins for ubiquitylation is determined by variable substrate binding of F-box proteins that interact with Skp1, as well as with Cul1. The assembly and activity of SCF ligases is regulated by the ligation of the ubiquitin-like protein NEDD8 to a specific lysine residue at the C-terminal domain of Cul1 [1, 3]. Neddylation of Cul1 increases the activity of SCF ligases by enhancing their affinity for E2 enzymes [4], and also regulates the assembly of SCF ligases by preventing Cul1 reassembly to the structural-based inhibitory protein CAND1 [5–8]. Structural insight into the mechanism by which neddylation mediates its effect on CRLs indicated that it causes a conformational change that eliminates the CAND1-binding site. On the other hand neddylation induces the RING finger protein flexibility which probably stimulates the CRL activity [9]. Previous work demonstrated that dissociation of CAND1 from its tightly bound cullin protein is enabled by F-box proteins such as Skp2 [10].

The extent of cullin protein neddylation is determined by a steady state of 2 opposite processes. Neddylation itself is conducted through an enzymatic cascade involving the E1-like enzyme APP-BP1/UBA3 and the E2-like enzyme UBC12. Removing the NEDD8 molecule, a process called deneddylation, is conducted by the COP9/Signalosome (CSN) complex. CSN is an 8-subunit complex that was highly conserved through evolution. The specific isopeptidase activity of the CSN5 subunit catalyzes deneddylation [11, 12]. The role of CSN in a number of human cancers, through overproduction of its CSN5 subunit, is well established [13].

To date, very little is known about the regulation of deneddylation activity of CSN. The cyclin-dependent kinase inhibitor p27 degradation cascade demonstrated in vitro direct co-interaction with CSN activity to deneddylate Cul1 and vice versa. In this model, a protein complex that included phosphorylated p27, cyclin E and CDK2—along with a partially need for the adaptor protein CKS1—prevented the action of CSN to deneddylate Cul1 [10]. Many substrates were shown to interact with CSN5 or with other subunits of the CSN. F-box protein Fbw7 complexed with Skp1 was also shown to inhibit deneddylation. This inhibitory effect was substantially increased upon addition of the phospho-cyclin E-CDK2 substrate [14]. Inhibition of deneddylase activity of CSN was also reported to be mediated by the E2 enzyme CDC34, and to a lesser extent by UBCH5 [14].

CSN demonstrated in vitro catalytic inhibition of CRL activity, which was due to its deneddylase activity [15]. CSN was also shown to inhibit unmodified SCF ligase ubiquitylation activity in a non-catalytic mechanism. This mode of inhibition was attributed to the stable bond that is formed between the CSN and cullin proteins, regardless of the NEDD8 modification status of the latter [14].

Taken together, the mechanism of CSN regulation is not yet understood. The high conservation of CSN in evolution suggests that a mechanism for the regulation of CSN deneddylase activity may exist. In this work we show that the rate of Cul1 deneddylation by CSN is determined by a simultaneous neddylation process. The fact that even a minor fraction of neddylated Cul1 has a substantial role in the regulation of SCF ligase activity attests to the physiologic importance of our findings. We hereby suggest a mechanism by which neddylation and deneddylation are engaged. We also found that intrinsic phosphorylation of CSN has no part in the regulation of the CSN deneddylase activity.

## Results

### COP9/Signalosome (CSN) mediates specific deneddylase activity

**Fig. 1:**
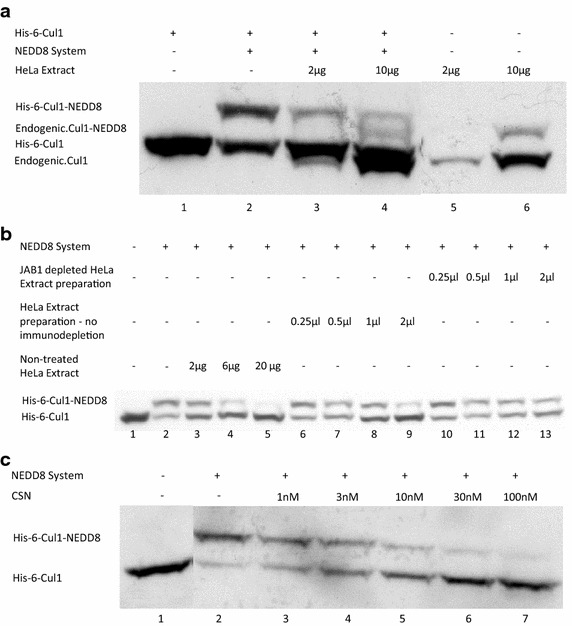
Cul1 deneddylation is mediated specifically by CSN. **a** 1 nM His-6-Cul1/ROC1 was pre-neddylated for 30 min at 30°C; and for 20 min more, at 30°C, following the addition of elevated volumes of HeLa cells extract. *Lanes* *5*–*6* represent equivalent volumes of HeLa cells extract blotted with anti-Cul1 antibody; this compares the resultant fraction of endogenic Cul1 with its neddylated conjugate, to the extract blotted without the addition of His-6-Cul1/ROC1. Same deneddylation activity was achieved while using reticulocyte lysates as a source for COP9/Signalosome under the same conditions; the image was cropped since most of the work was conducted with HeLa cells extract due to technical reasons and the complete blot is provided as an additional data file termed Additional file 1. **b** 10 nM His-6-Cul1/ROC1 were pre-neddylated for 30 min at 30°C, followed by the addition of elevated volumes of HeLa cells extract, and by preparations that were either immunodepleted from JAB1 or that had undergone the same procedure without the antibody itself, as discussed in the methods section. The incubation time with the extract and its parallel derivatives was 20 min at 30°C. **c** 3 nM His-6-Cul1/ROC1 was pre-neddylated for 30 min at 30°C, followed by the addition of an elevated concentration of purified CSN for 10 min at 20°C. The first part of the image was cropped since it tested different conditions for neddylation and is not relevant to the data exemplified regarding the deneddylase activity of the purified CSN. The complete blot is provided as an additional data file termed Additional file 2.

As an initial step we set an in vitro deneddylation reaction in order to identify an isolated and specific CSN activity that deneddylates Cul1. We first identified endogenic CSN activity using HeLa cells extract incubated in elevated volumes with preneddylated recombinant Cul1 (Fig. 1a). To demonstrate that deneddylation is performed specifically by CSN, we used a specific antibody against the catalytic subunit of CSN (Jab1/CSN5). Using cell extract that was immunodepleted of Jab1 in a deneddylation reaction system did not decrease the neddylated fraction of Cul1 compared to that obtained using an extract subject to the same procedure without the immunodepletion of Jab1 (Fig. 1b, compare lanes 6–9 with lanes 10–13). Purified CSN demonstrated increased deneddylase activity, while elevating its reaction concentration, the same as was shown when using endogenic CSN (Fig. 1c).

### Depletion of ATP inhibits the CSN mediated deneddylation reaction rate

To isolate the deneddylation reaction from its opposite and parallel neddylation reaction we pre-neddylated recombinant Cul1 and then added hexokinase with its 2-d-glucose substrate, which depletes ATP from the original reaction mix. This ATP depleting reaction will be termed from here on as: “ATP-trap”. Depleting ATP enabled examination of the rate of deneddylation without interference from the opposite process of neddylation, with its influence on the final percentage of neddylated Cul1.

**Fig. 2:**
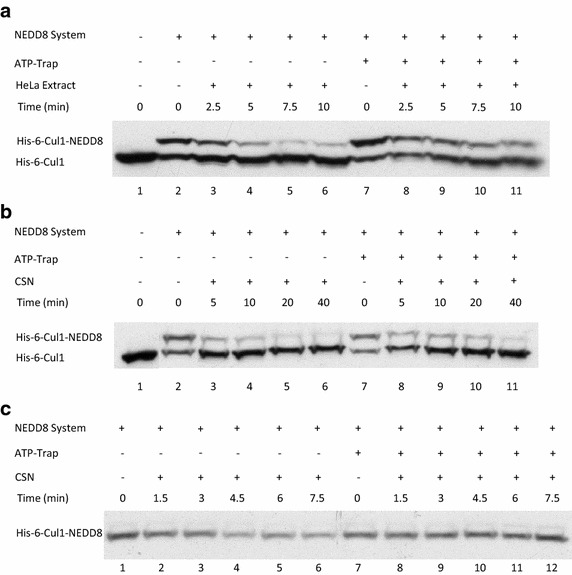
ATP-depletion resulted in the inhibition of CSN deneddylase activity. **a** 10 nM His-6-Cul1/ROC1 were pre-neddylated for 30 min at 30°C. Then 10 μg HeLa cells extract were added at 30°C (time plot: *lanes* *3*–*6*); or alternatively, ATP-trap mix was added for 45 min at 30°C, followed by the addition of 10 μg HeLa cells extract at 30°C (time plot: *lanes* *8*–*11*). **b** 3 nM His-6-Cul1/ROC1 was pre-neddylated for 30 min at 30°C. Then 100 nM purified CSN was added at 20°C (time plot: *lanes 3*–*6*); or alternatively, ATP-trap mix was added for 45 min at 30°C, followed by the addition of 100 nM purified CSN at 20°C (time plot: *lanes 8*–*11*). **c** 10 nM His-6-Cul1/ROC1 was incubated as described in **b**; time plots were for 1.5–7.5 min, and not for 5–40 min as in **b**. Blotting was directed against NEDD8, and not against Cul1, as described elsewhere.

First, we set up a neddylation reaction. At this stage, we either added or did not add an ATP-trap system. Then, we added HeLa cells extract on a time plot, while measuring the rate of deneddylation. Surprisingly, we discovered that depleting ATP from the reaction mix resulted in inhibition of the deneddylation reaction rate (Fig. 2a). When we examined the same process using only purified and recombinant factors in the reaction, we noted that adding an ATP-trap system followed with the addition of purified CSN inhibited deneddylation similar to the effect that addition of cell extract to the reaction yielded (Fig. 2b). We next used an anti-NEDD8 antibody in order to focus on the quantitative differences at the level of neddylated Cul1, while both neddylation and deneddylation occur simultaneously; or while deneddylation occurs separately, without parallel neddylation (Fig. 2c). The same inhibitory effect upon deneddylation of Cul1 was observed when the two processes were isolated by adding the ATP-trap system. Quantitative curves representing the results examplified in Fig. 2a–c are provided as additional files named Additional file 3: Graph 2A, Additional file 4: Graph 2B, Additional file 5: Graph 2C respectively.

### CSN deneddylase activity is not mediated through intrinsic phosphorylation

**Fig. 3:**
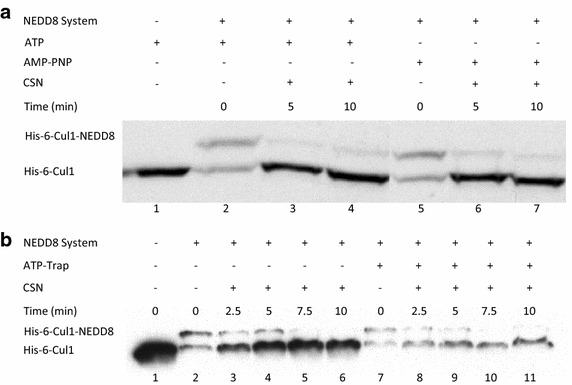
Reconstitution of ATP with AMP-PNP does not inhibit Cul1 deneddylation. **a** 3 nM His-6-Cul1/ROC1 was pre-neddylated for 30 min at 30°C, with either ATP or AMP-PNP, then followed with an addition of 100 nM purified CSN, on a time plot of 5–10 min at 20°C. Part of the image was cropped since the experiment also tested the use of CSN after the addition of ATP-trap mix with either ATP or AMP-PNP as was exemplified and discussed in Fig. 2 (regarding the use of ATP). Those parts of the experiment are not relevant for the question of CSN hypothetic autophosphorylation. The complete blot is provided as an additional data file termed Additional file 6. **b** 10 nM His-6-Cul1/ROC1 was pre-neddylated for 30 min at 30°C with AMP-PNP. Then 100 nM purified CSN was added at 20ºC (time plot: *lanes 3*–*6*); or alternatively, ATP-trap mix was added for 45 min at 30°C, followed by the addition of 100 nM purified CSN at 20°C (time plot: *lanes* *8*–*11*).

The presence of ATP in the reaction mix resulted in an enhanced rate of Cul1 deneddylation. This inspired examination of whether phosphorylation of CSN mediates, at least in part, the regulation of deneddylation. The potential of CSN units to undergo phosphorylation, with its hypothetical physiologic importance, is well established. Likewise, a potential kinase activity is known to exist by associated protein kinases, such as casein kinase 2 (CK2), inositol 1,3,4-triphosphate 5/6 kinase, and protein kinase D (PKD), which are purified along with the CSN itself [16–18]. Reconstitution of ATP with AMP-PNP enables neddylation, by releasing the pyrophosphate that is needed for the reaction, yet no phosphorylation can occur. After pre-neddylation of recombinant Cul1 with either ATP or AMP-PNP, we added to the reaction purified CSN. This was to assess the hypothetic difference in the rate of deneddylation when intrinsic phosphorylation is not possible. The result was that no difference at all was detected between the two reactions (Fig. 3a, compare lanes 3–4 with lanes 6–7). In order to further define that the use of AMP-PNP is not influenced by the ATP-trap system we conducted 2 time plots for deneddylation catalyzed by purified CSN without or after adding an ATP-trap to the reaction. As can be noted in Fig. 3b the rate of Cul1 deneddylation is not influenced by the addition of the ATP-trap ingredients while using AMP-PNP as the source for the pyrophosphate group that is catalyzed during neddylation (compare lanes 3–6 with lanes 8–11).

### Deneddylation rate is enhanced when occurring in parallel to the neddylation reaction

**Fig. 4:**
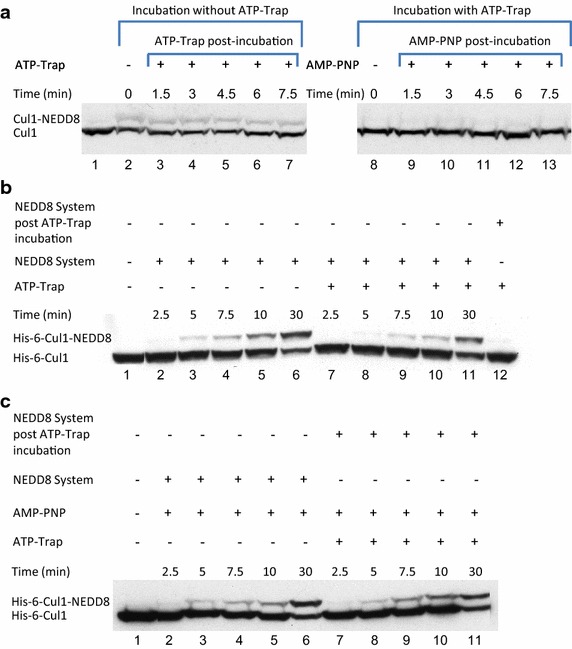
Endogenic Cul1 deneddylation is enhanced during simultaneous neddylation. **a** 20 μg HeLa cells extract were incubated for 30 min at 30°C (*lanes 2*–*7*) or with the addition of ATP-trap mix prior to the incubation (*lanes 8*–*13*). Simultaneous ATP-depletion was performed in *lanes 3*–*7* after 30 min incubation, on a time plot of 1.5–7.5 min at 30°C. Simultaneous neddylation was performed in *lanes 9*–*13* after 30 min, by the addition of AMP-PNP, on a time plot of 1.5–7.5 min at 30°C. **b** 10 nM His-6-Cul1/ROC1 was neddylated at 30°C on a time plot of 2.5–30 min, without or with the addition of ATP-trap mix (*lanes 2*–*6* and *lanes 7*–*11*, respectively). In *lane 12*, the neddylation mix was added to the reaction after pre-incubation with ATP-trap mix. **c** 10 nM His-6-Cul1/ROC1 was neddylated at 30°C using AMP-PNP on a time plot of 2.5–30 min, either directly (*lanes 2*–*6*) or after pre-incubation with ATP-trap mix (*lanes 7*–*11*).

To identify the mechanism by which the presence of ATP promotes deneddylation, we modified the conditions of the steady state formed between the two opposing reactions (neddylation versus deneddylation) on endogenic Cul1 in HeLa cells extract. Preliminary experiments identified that endogenic ATP exists in the extract and enables the neddylation reaction to modify the endogenic Cul1 with NEDD8, even without the addition of exogenic ATP or recombinant neddylation enzymes (e.g. APP-BP1/UBA3 and UBC12) and NEDD8 itself (data not shown). We compared the steady state of the endogenic Cul1 neddylation formed either when the extract is incubated alone or when an ATP-trap system is added at the beginning of the reaction (Fig. 4a, compare lane 2 with lane 8). As can be seen, the addition of the ATP-trap system at the beginning of the reaction prevented even minor neddylation to occur. This demonstrates the high potency of CSN deneddylase activity in these conditions. On the other hand, a time plot of HeLa cells extract to which the ATP-trap system was added after a steady state was reached, showed no effect on endogenic Cul1 deneddylation (Fig. 4a, lanes 3–7). This observation contrasts with the expectation that the simultaneous depletion of ATP from the reaction would cause the neddylation rate to diminish and thus be exceeded by the rate of deneddylation. Neddylation of recombinant Cul1 in the presence of the simultaneous depletion of ATP proved to substantially decrease the degree of Cul1 neddylation, even in small time intervals, as shown in Fig. 4b (compare lanes 2–6 with lanes 7–11).

To examine the performance of a new steady state of endogenic Cul1 neddylation status in the presence of simultaneous neddylation and deneddylation reactions, we conducted a time plot of the addition of AMP-PNP to the extract that was pre-incubated with the ATP-trap system. AMP-PNP overcomes the hexokinase effect to deplete ATP since it does not serve as a substrate for the hexokinase reaction with 2-d-glucose, as shown in Fig. 4c (equal Cul1 neddylation rate with either the addition of ATP-trap system or without it, compare lanes 2–6 with lanes 7–11 in Fig. 4c). This contrasts with the absolute prevention of neddylation when the reaction mix containing ATP is pre-incubated with the ATP-trap system, and the neddylation enzymes are only then added (Fig. 4b, lane 12). The addition of AMP-PNP to the extract that was pre-incubated with an ATP-trap system clearly did not shift the rate of Cul1 neddylation status (Fig. 4a, lanes 9–13). The resultant rate of simultaneous deneddylation was substantially elevated from its rate when the reaction was conducted separate from neddylation, as demonstrated in the first part of the experiment and discussed above (Fig. 4a, lanes 3–7).

### Direct inhibition of neddylation with MLN-4924 diminishes the rate of deneddylation

**Fig. 5:**
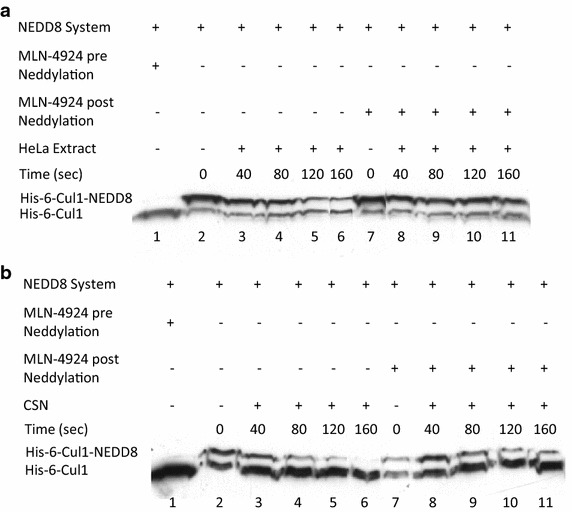
Direct inhibition of APP-BP1/UBA3 with MLN-4924 results in inhibition of CSN deneddylase activity. **a** 10 nM His-6-Cul1/ROC1 was pre-neddylated for 30 min at 30°C. Then 10 μg HeLa cells extract was added, on a time plot at 20°C (*lanes 3*–*6*); or, alternatively, MLN-4924 was added for 15 min at 30°C, followed by the addition of 10 μg HeLa cells extract, on a time plot at 20°C (*lanes 8*–*11*). In *lane 1*, incubation with MLN-4924 was prior to neddylation. **b** The same as **a** using 100 nM purified CSN instead of HeLa cells extract. Both images 5a, b were cropped and the *lanes* representing the end of the time plot (200 s) were omitted due to technical reasons. The complete blots are provided as additional data files termed Additional file 9 and Additional file 10, respectively.

MLN-4924 is a selective inhibitor of the NEDD8 activating enzyme APP-BP1/UBA3 [19]. We used MLN-4924 to demonstrate a direct shift in the rate of deneddylation, resulting from direct inhibition of neddylation. MLN-4924 was incubated with pre-neddylated Cul1 and deneddylation was initiated on a time plot using HeLa cells extract (Fig. 5a) or purified CSN (Fig. 5b). The rates of deneddylation were compared between an experiment that was simultaneously conducted with neddylation and an experiment in which deneddylation was isolated after MLN-4924 preincubation (Fig. 5a, b, compare lanes 3–6 with lanes 8–11). As can be clearly seen, direct inhibition of neddylation resulted in subsequent inhibition of the deneddylation rate. It should be emphasized that the addition of MLN-4924 to the reaction resulted in definite and complete inhibition of Cul1 neddylation, as demonstrated in lane 1 of Fig. 5a, b. Quantitative curves representing the results examplified in Fig. 5a, b are provided as additional files named Additional file 7: Graph 5A, Additional file 8: Graph 5B respectively.

### p27 ubiquitylation is linearly enhanced in minimal SCF^SKP2^ neddylation status

**Fig. 6:**
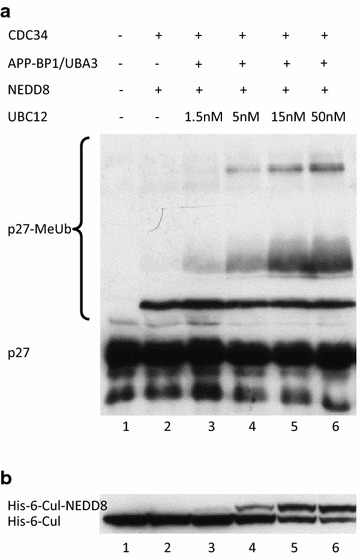
Linear elevation of the Cul1-neddylation fraction promotes linear elevation in p27-ubiquitin conjugated fraction. His-6-p27 was conjugated to methylated ubiquitin for 60 min at 30°C as described above, and with elevated concentrations of UBC12. Blotting was directed against p27 (**a**) or Cul1 (**b**).

In order to examine the importance of the findings described above we conducted an assay that tests the influence of changes in Cul1 neddylation fraction upon the degree of p27 conjugation. We performed a p27 conjugation reaction with elevated concentrations of UBC12 and assessed the linear catalytic rate of the reaction. The minor Cul1 neddylation fraction proved to substantially increase p27 conjugation to methylated ubiquitin. Moreover, linear increase of neddylation resulted in linear increase of p27 ubiquitylation (Fig. 6). These findings further demonstrate the biochemical effect of changes in neddylation/deneddylation balance upon physiologic intracellular processes, such as tumor suppressor p27 ubiquitylation and other target substrates of CRL ligases.

## Discussion

The findings of this work suggest that deneddylation of Cul1 is an enzymatic process, with a rate of reaction that is dependent upon the opposing process of neddylation. Already one decade ago, it was hypothesized that cycles of neddylation and deneddylation enable cells to precede through the cell cycle [20, 21–22]. Our findings directly demonstrate shifts in the steady state that is established between neddylation and deneddylation, and these changes are dependent upon the active state of neddylation. The current study showed that simultaneous active neddylation promotes CSN deneddylase activity. Our findings hypothetically contrast with Soucy et al’s report that application of the NEDD8 conjugation inhibitor MLN-4924 to cells results in rapid loss of NEDD8 conjugates [19]. It should be emphasized, however that all our results and their biochemical interpretation refer to an in vitro reaction while Soucy’s findings are based on using intact cells. On the other hand Brownell et al. [23] detected recovery of neddylation in cells following the initial effect of MLN-4924 towards rapid and preliminary deneddylation. This observation hints for the possibility of an in vivo late reactant reduction in deneddylating activity, which is consistent with our results and hypothesis.

Structural changes provide a plausible mechanism for the shifts in the neddylation steady state, which result from the ATP-depleting reaction and from direct neddylation inhibition. Zheng et al. reported that ATP markedly promotes dissociation of Cul1 from CAND1 [6]. This observation might have influenced our results and their interpretation yet a few factors relevant to our assay contradict the hypothetic technical influence of ATP depletion from the reaction: first, the same findings were demonstrated either while using cells extracts or while using recombinant reagents without adding any hypothetical source for endogenic CAND1. Second, in Zheng’s work elevated ATP concentrations in the reaction correlated with higher neddylated Cul1 fraction yet the initial lowest ATP concentration that promoted the CAND1-Cul1 complex to dissociate was 2 mM. In our assay we used 0.5 mM ATP which definitely couldn’t be relevant to any hypothetic interference with our assay. Third, the use of MLN-4924 to directly inhibit Cul1 neddylation is not dependent upon the use of ATP. Its use promoted the same significant trend towards inhibition of Cul1 deneddylation that was demonstrated while using the ATP-trap modification. Another technical issue that had to be tackled was the fact that depleting ATP from the reaction might have influenced its pH. The use of 50 mM Tris–HCl (pH 7.6) as our reaction’s buffer maintains constant pH during all phases of the reaction regardless of either depleting ATP or adding the ATP-trap system.

Our hypothesis of the mechanism involved relies on the high affinity that demonstrated between CSN and Cul1, regardless of the latter’s state of neddylation. It seems reasonable that structural changes in Cul1 during its neddylation reaction govern its parallel availability for efficient deneddylation. This hypothesis may also rely on the evidence that CSN forms supercomplexes with CRLs as well as with larger particles such as the 26S proteasome [24–29]. The formation of such supercomplexes might serve as a conformational platform that enables regulated and synchronized cycles of neddylation and deneddylation. The fact the mentioned above CSN precipitates contain in most of the cases neddylated cullins also hint for our hypothesis.

Another hypothetical explanation for our findings is the fact that deneddylated Cul1 accumulates and changes the steady state of the reactions neddylation/deneddylation. The accumulation of unmodified Cul1 might inhibit CSN activity. Emberley et al. demonstrated that incubation of elevated unneddylated Cul1 concentrations resulted in a significant reduction of CSN activity. However, the incubation conditions in that study related to a simultaneous neddylation/deneddylation enzymatic reaction, and not to an isolated deneddylation reaction [14].

Relevant to the cyclic pattern of the CRL mode of action, is the important observation that the stoichiometric connection that has been shown between CAND1 and Cul1 is interrupted by F-box proteins such as Skp2 [10]. This initial interrupting phase is further enhanced due to Cul1 neddylation. The cascade of events induces an active state of CRL. On the other hand, the accumulation of substrates in their pre-conjugated state (e.g complexed with adaptor proteins and phosphorylated) inhibits deneddylation. This in turn further activates their catalytic state, due to additional neddylation. The current work concurs with previous studies that demonstrated that linear elevation in the neddylated fraction of Cul1 results in linear elevation in the conjugated fraction of substrates such as p27. The process described above has its own opposing effect, which results from the fact that CRL also mediates auto-conjugation and subsequent degradation of the intra-components of the ligase itself, such as the F-box protein. Skp2, for example undergoes auto-ubiquitylation [30] and Cul1 neddylation promotes this process (data not shown). The result is destabilization of the ligase structure and the possible promotion of the modulation of CRL towards the formation of parallel SCF complexes, or the silencing of CRL by promoting the recovery of Cul1-CAND1 bonding. The core regulation that governs the stability of CRL may be assumed to rely on the cycles described above, between neddylation and deneddylation. Structural changes in the position of Cul1 towards its 2 opposing modifying enzymatic reactions may indeed explain the utility of these processes and the need for their simultaneous biochemical crosstalk.

In this work we also tried to analyze the hypothetic role of phosphorylation in the regulation of CSN. The use of AMP-PNP enables us to catalyze Cul1 neddylation due to its pyrophosphate group which can be utilized by the nedd8 E1 enzyme APP-BP1/UBA3. On the other hand AMP-PNP can’t contribute a free phosphate group hence it can’t play a hypothetic role in CSN subunints or adaptor proteins phosphorylation. As can be noticed in Fig. 3 the use of both ATP and AMP-PNP elicited a preneddylation of Cul1. Addition of CSN to the reaction resulted in both cases with a rapid and significant deneddylation.

It is well established that deneddylation by itself has no prerequisite for ATP yet it was tempting to hypothesize that phosphorylation might regulate deneddylation. The fact that AMP-PNP had no effect on the rate of CSN deneddylase activity rules out the hypothesis that deneddylation might be regulated by either autophosphorylation or adaptor proteins kinases activity. The observation that intrinsic CSN phosphorylation does not have a role in the regulation task of deneddylase activity raises again the question of whether other factors might regulate CSN. Previous works demonstrated phosphorylation of CSN and showed that phosphorylation of the CSN1 subunit is necessary for efficient assembly of the CSN into a ß-catenin degrading supercomplex [25]. Despite the lack of involvement of CSN associated kinases in direct regulation of deneddylation, it is tempting to speculate that the targeting of substrates by phosphorylation might have a regulatory effect on CSN deneddylase activity.

A recently published study demonstrated that the manner that CSN5 integrates in the CSN complex probably provides the conformational energy that enables deneddylation to occur, and that CSN5 in its CSN independent form lacks the ability to connect its NEDD8 substrate and to perform its catalytic reaction [31].

Elucidation of the biochemical mechanisms that regulate CSN activity is important for the pharmacologic targeting of the NEDD8 system components that have started to emerge [19]. Since CSN itself seems to play a definite role in tumor pathogenesis, understanding its fine mode of action and regulation will provide a solid platform in developing pharmacologic solutions.

## Conclusions

Cul1 deneddylation is an intracellular enzymatic process which is catalyzed by the COP9/Signalosome. This complex is highly conserved through evolution. In this work we demonstrated that deneddylation is positively regulated through simultaneous neddylation. We were able to do so through some levels of proof using different modes to inhibit neddylation either by depleting ATP from the reaction or by directly inhibit neddylation using its synthetic inhibitor MLN-4924.

Regarding the regulation of deneddylation we were also able to demonstrate that auotophophrylation of COP9/Signalosome probably doesn’t govern its catalytic activity.

The significance of our findings is emphasized by the fact that minor changes in the degree of Cul1 neddylation linearly control the degree of p27 conjugation to ubiquitin, a process known to have cardinal effect towards the regulation of cell cycle.

## Methods

### Reagents

His-6-Cul1/ROC1, His-6-APP-BP1/UBA3, His-6-Skp1/Skp2, and His-6-Cyclin E/CDK2 were produced by co-infection of 5B insect cells with baculoviruses encoding the corresponding proteins, and were purified by nickel-agarose chromatography as previously described [33]. His-6-p27, His-6-UBC12 and His-6-CDC34 were bacterially expressed and purified by chromatography on nickel-agarose. NEDD8 was bacterially expressed and purified as previously described [32]. Bacterially expressed Cks1 was purified as previously described [33]. E1, HeLa cells extract and methylated ubiquitin were kindly provided by A Hershko (Technion-Israel Institute of Technology, Haifa, Israel). Purified CSN from human erythrocytes was purchased from Enzo Life Sciences catalog no. BML-PW9425-0010. MLN-4924 was purchased from Active Biochem catalog no. A-1139.

The following antibodies were used for immunoblotting and immunodepletion: anti-Cul1 rabbit polyclonal antibody, Invitrogen catalog no. 71-8700; anti-NEDD8 rabbit polyclonal antibody, Invitrogen catalog no. 34-1400; anti-p27 mouse monoclonal antibody, BD Transduction Laboratories catalog no. 610242; anti-Jab1 goat polyclonal antibody, Santa Cruz Biotechnology catalog no. sc-6271; and anti-JAB1 mouse monoclonal antibody, Santa Cruz Biotechnology catalog no. sc-135954.

### In-vitro neddylation reaction

His-6-Cul1/ROC1 was incubated in the presence of 300 nM NEDD8, 50 nM UBC12, 2 nM APP-BP1/UBA3, 50 mM Tris–HCL (pH 7.6), 5 mM MgCl_2_, 1 mM DTT, 3 mg/ml ovalbumin, 10% glycerol, 10 mM phosphocreatine, 0.1 μg/μl creatine phosphokinase, and either 0.5 mM ATP or 0.5 mM AMP-PNP, unless stated otherwise. HeLa cells extract was incubated without the addition of neddylation enzymes and NEDD8, nor the addition of ATP. The reaction volume was 10 μl, and incubation time was 30 min in 30°C. Neddylation was terminated either by adding SDS buffer or by continuing with the deneddylation reaction as discussed below.

### In-vitro deneddylation reaction

Pre-neddylated His-6-Cul1/ROC1 was incubated with either HeLa cells extract as a source of endogenic CSN, or with 100 nM purified CSN. The deneddylation rate was tested in 3 modifications: (1) directly/simultaneously after Cul1 pre-neddylation, (2) after incubation for 45 min of pre-neddylated Cul1 in 30°C with ATP-trap mix, which included 20 mM 2-d-glucose and 1 μg/μl hexokinase, (3) after incubation for 15 min of pre-neddylated Cul1 in 30°C with 50 μM MLN-4924.

Endogenic Cul1 deneddylation was tested after incubating HeLa cells extract in the presence of the ATP-trap system. This was carried out without preincubation and without time plot format incubation, subsequent to the completion of neddylation/deneddylation steady state incubation, as discussed above. Purified CSN was not added to the HeLa cells extract containing reactions.

### p27 ubiquitylation reaction

50 nM His-6-p27 was incubated with elevated concentrations of UBC12 and in the presence of 1 pmol E1, 3 μM CDC34, 10 nM His-6-Skp1/Skp2, 20 nM His-6-Cul1/ROC1, 20 nM His-6-Cyclin E/CDK2, 100 nM Cks1, 300 nM NEDD8, 1 nM APP-BP1/UBA3, 50 mM Tris–HCL (pH 7.6), 1 mg/ml methylated ubiquitin, 5 mM MgCl_2_, 1 mM DTT, 3 mg/ml ovalbumin, 10% glycerol, 10 mM phosphocreatine, 0.1 μg/μl creatine phosphokinase, and 0.5 mM ATP. Reaction volume was 10 μl and incubation time was 60 min at 30°C.

### Jab1 immunodepletion assay

600 μg of HeLa cells extract was incubated, with or without anti-JAB1 goat polyclonal antibody, for 90 min at 30°C, and then rotated with protein G beads for 6 h at 4°C. The reaction volume was completed to a total of 50 μl, using phosphate-buffered-saline (PBS) and 3 μg/μl bovine serum albumin (BSA). The beads were washed with PBS and mixed with SDS buffer, to separate attached proteins while the supernatants underwent a second incubation with anti-JAB1, and rotation with protein G beads as detailed above. The final supernatants were diluted with a buffer of 50 mM Tris/1 mM DTT and reconcentrated to final volumes of 50 μl.

The supernatant preparations were immunoblotted using anti-JAB1 mouse monoclonal antibody. A specific complete JAB1 depletion was exemplified in the preparation originating from the anti-JAB1 antibody that was added to the mixture. This contrasted with the corresponding preparation, which was incubated originally without the specific antibody.

Elevated concentrations of the two preparations were tested in an in vitro deneddylation reaction as detailed above.

### Western blot

Reactions were terminated by the addition of SDS. Cul1, NEDD8, or p27 substrates were subjected to SDS-PAGE, transferred to nitrocellulose, and blotted with indicated antibodies. Immunoreactive bands were visualized with SuperSignal chemiluminescent reagent (Pierce) (Additional files 1, 2, 3, 4, 5, 6, 7, 8, 9, 10).

## Additional files

**Additional file 1.** Uncropped Fig. 1a.
**Additional file 2.** Uncropped Fig. 1c.
**Additional file 3.** Graph 2A.
**Additional file 4.** Graph 2B.
**Additional file 5.** Graph 2C.
**Additional file 6.** Uncropped Fig. 3a
**Additional file 7.** Graph 5A.
**Additional file 8.** Graph 5B.
**Additional file 9.** Uncropped Fig. 5a.
**Additional file 10.** Uncropped Fig. 5b.
