# Supplementary figures and images for: COP9-Signalosome deneddylase activity is enhanced by simultaneous neddylation: insights into the regulation of an enzymatic protein complex
Source: Cell Div. 2015 Aug 11;10:5. doi: 10.1186/s13008-015-0011-0 (PMC4531434; doi:10.1186/s13008-015-0011-0)

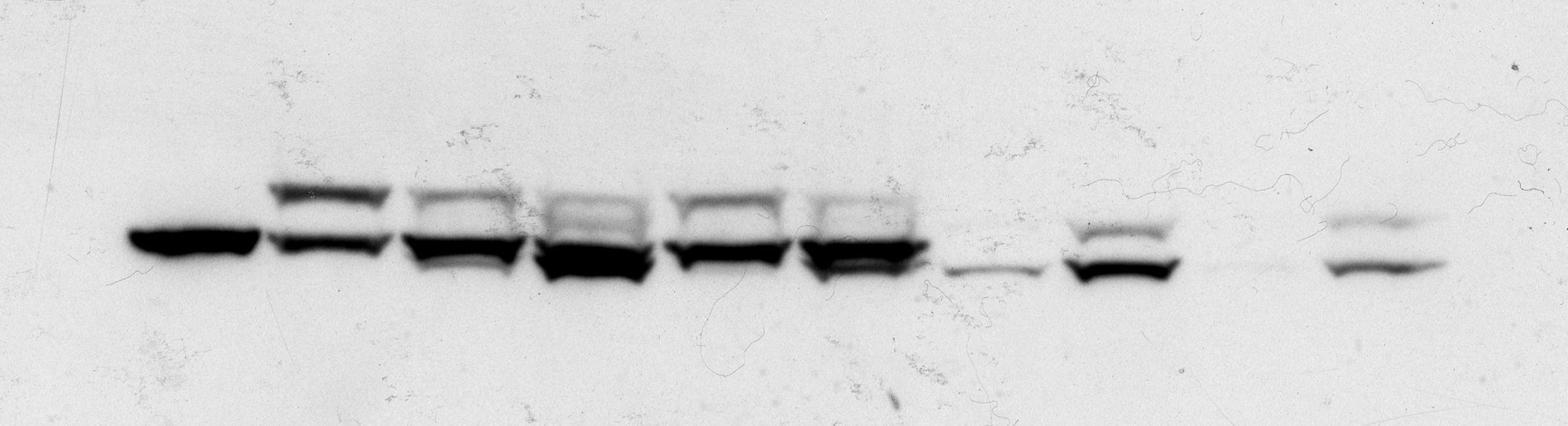

Supplement: Supplementary file 1 — Additional file 1. Uncropped Fig. 1a. [file 13008_2015_11_MOESM1_ESM.tiff]

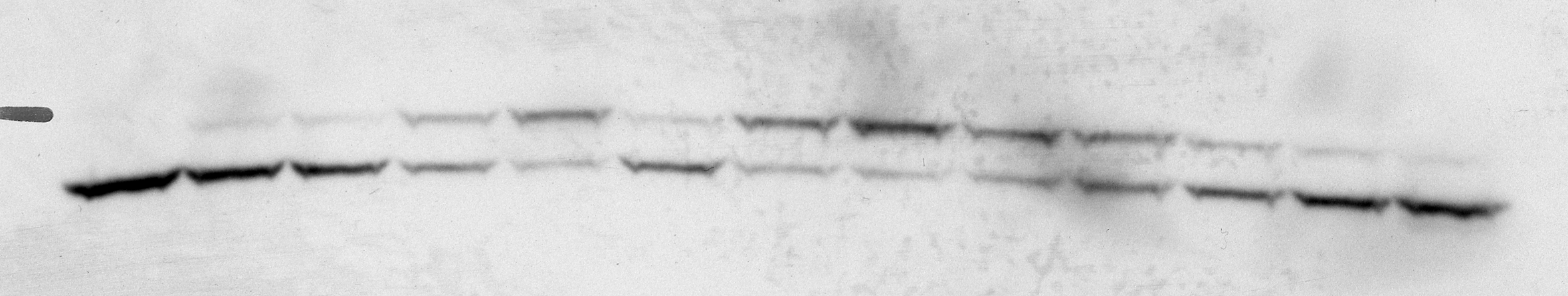

Supplement: Supplementary file 2 — Additional file 2. Uncropped Fig. 1c. [file 13008_2015_11_MOESM2_ESM.tiff]

## Slide 1
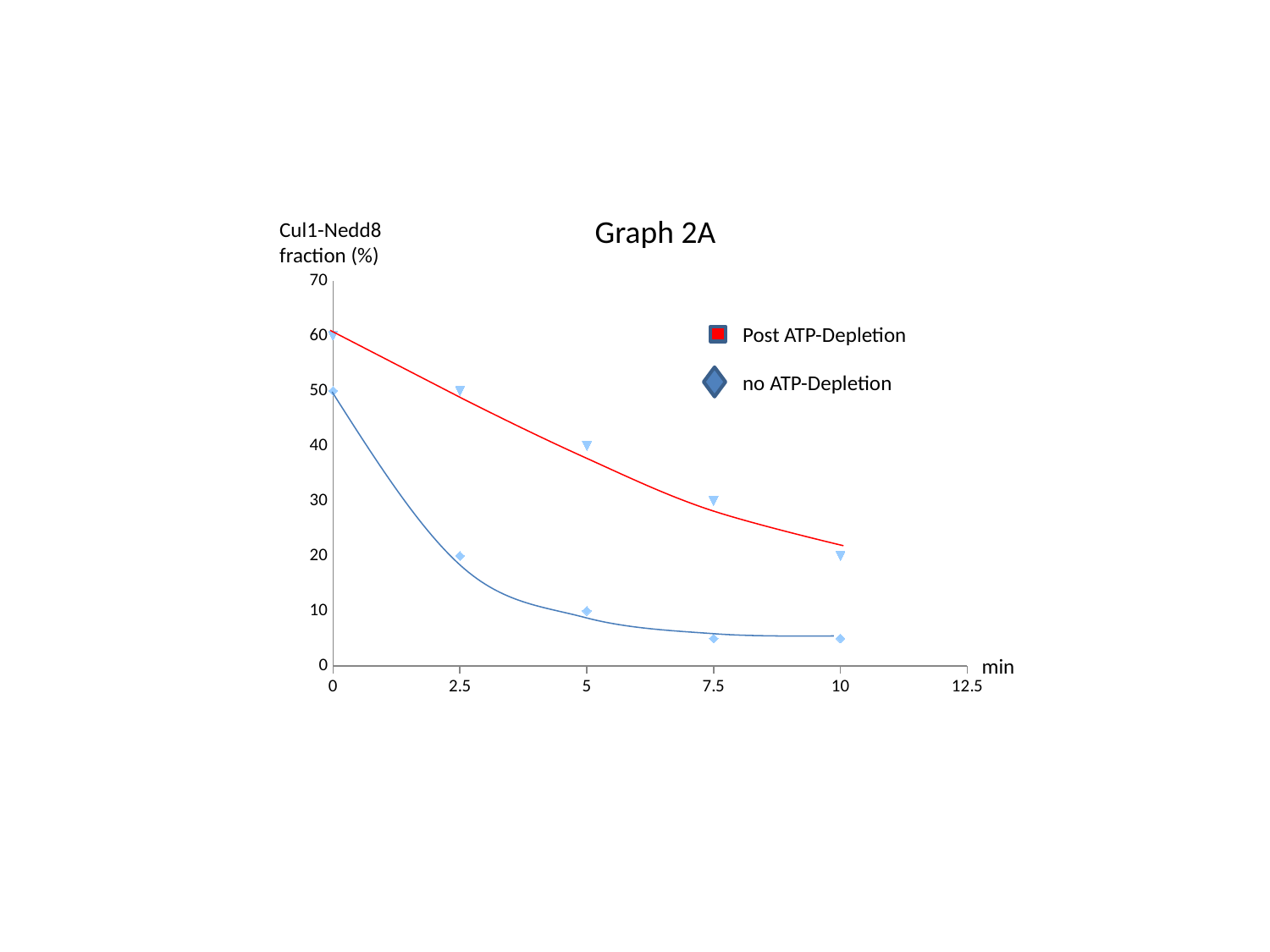

### Chart
| Category | | |
|---|---|---|
Cul1-Nedd8 fraction (%)
min
Graph 2A
Post ATP-Depletion
no ATP-Depletion

Supplement: Supplementary file 3 — Additional file 3. Graph 2A. [file 13008_2015_11_MOESM3_ESM.pptx]

## Slide 1
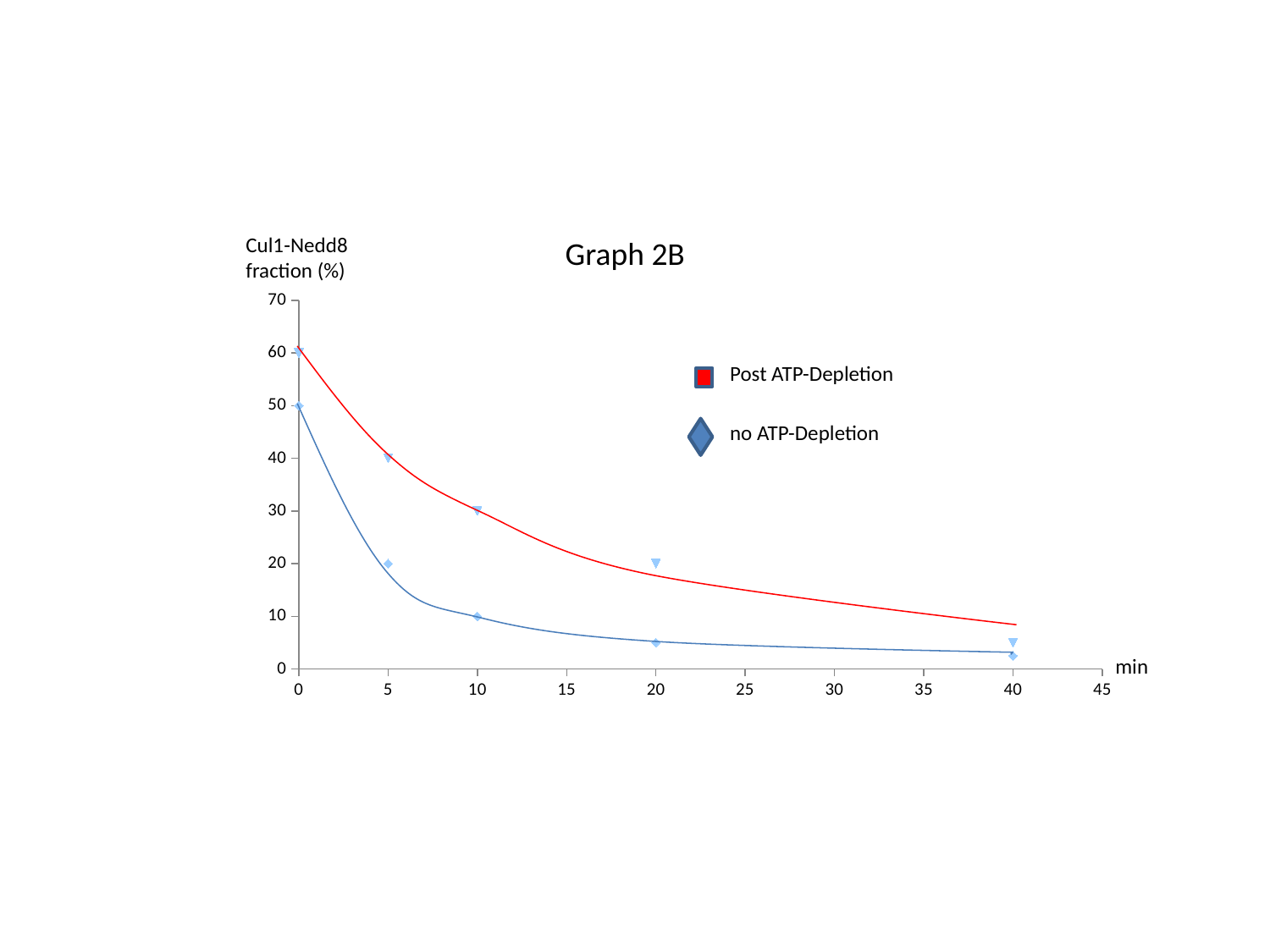

### Chart
| Category | | |
|---|---|---|Cul1-Nedd8 fraction (%)
Graph 2B
Post ATP-Depletion
no ATP-Depletion
min

Supplement: Supplementary file 4 — Additional file 4. Graph 2B. [file 13008_2015_11_MOESM4_ESM.pptx]

## Slide 1
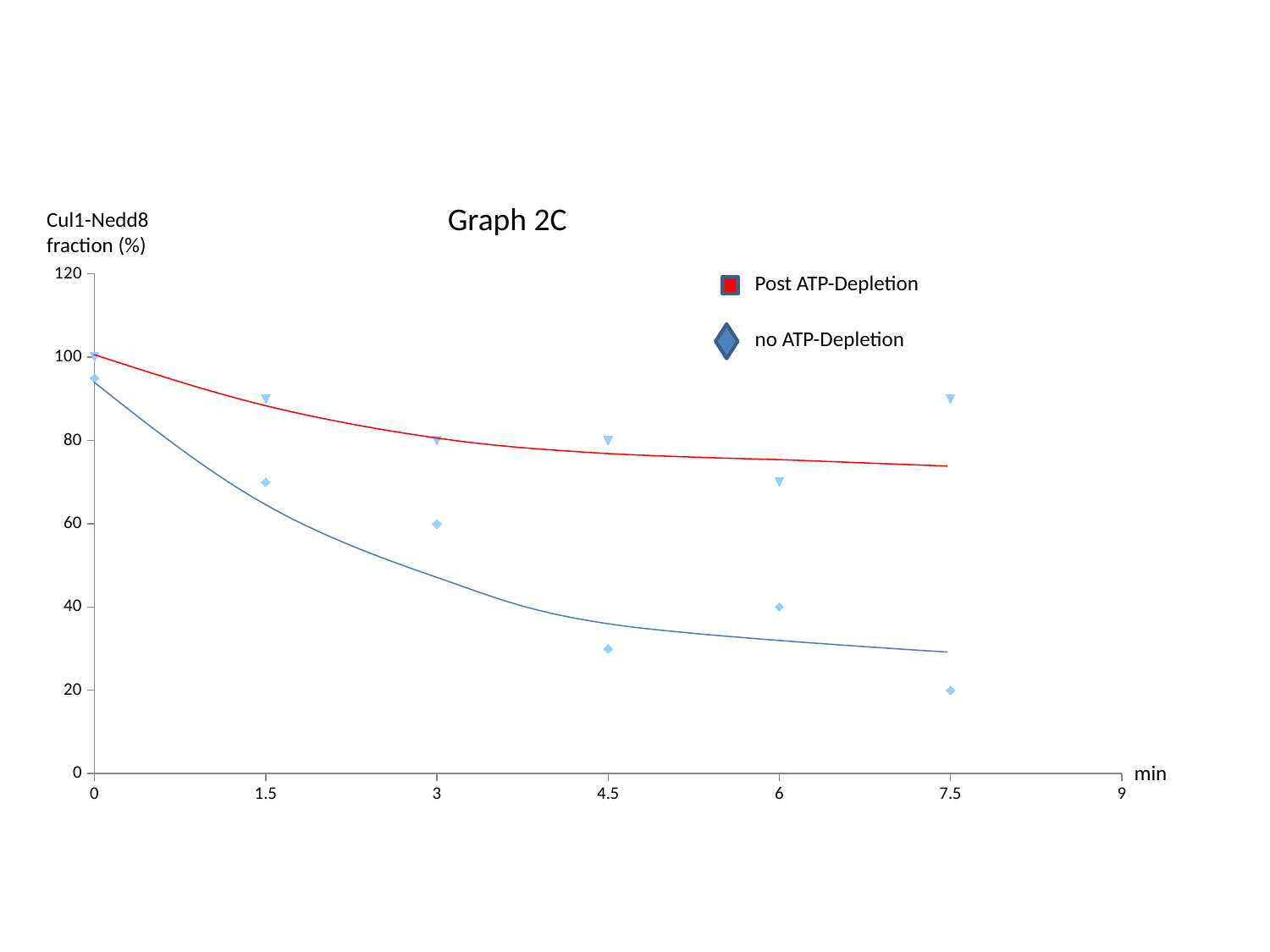

Graph 2C
Cul1-Nedd8 fraction (%)
### Chart
| Category | | |
|---|---|---|Post ATP-Depletion
no ATP-Depletion
min

Supplement: Supplementary file 5 — Additional file 5. Graph 2C. [file 13008_2015_11_MOESM5_ESM.pptx]

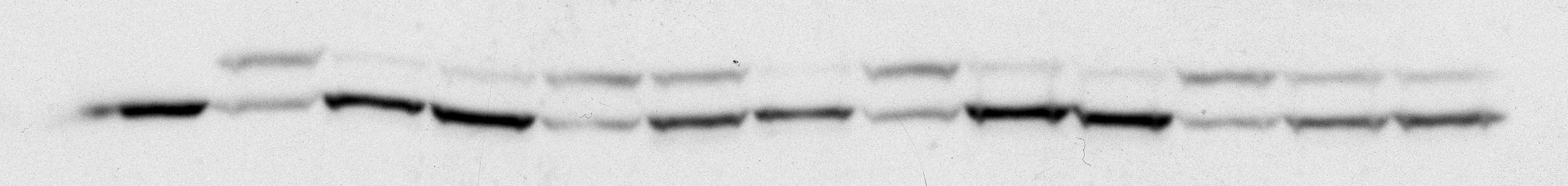

Supplement: Supplementary file 6 — Additional file 6. Uncropped Fig. 3a [file 13008_2015_11_MOESM6_ESM.tiff]

## Slide 1
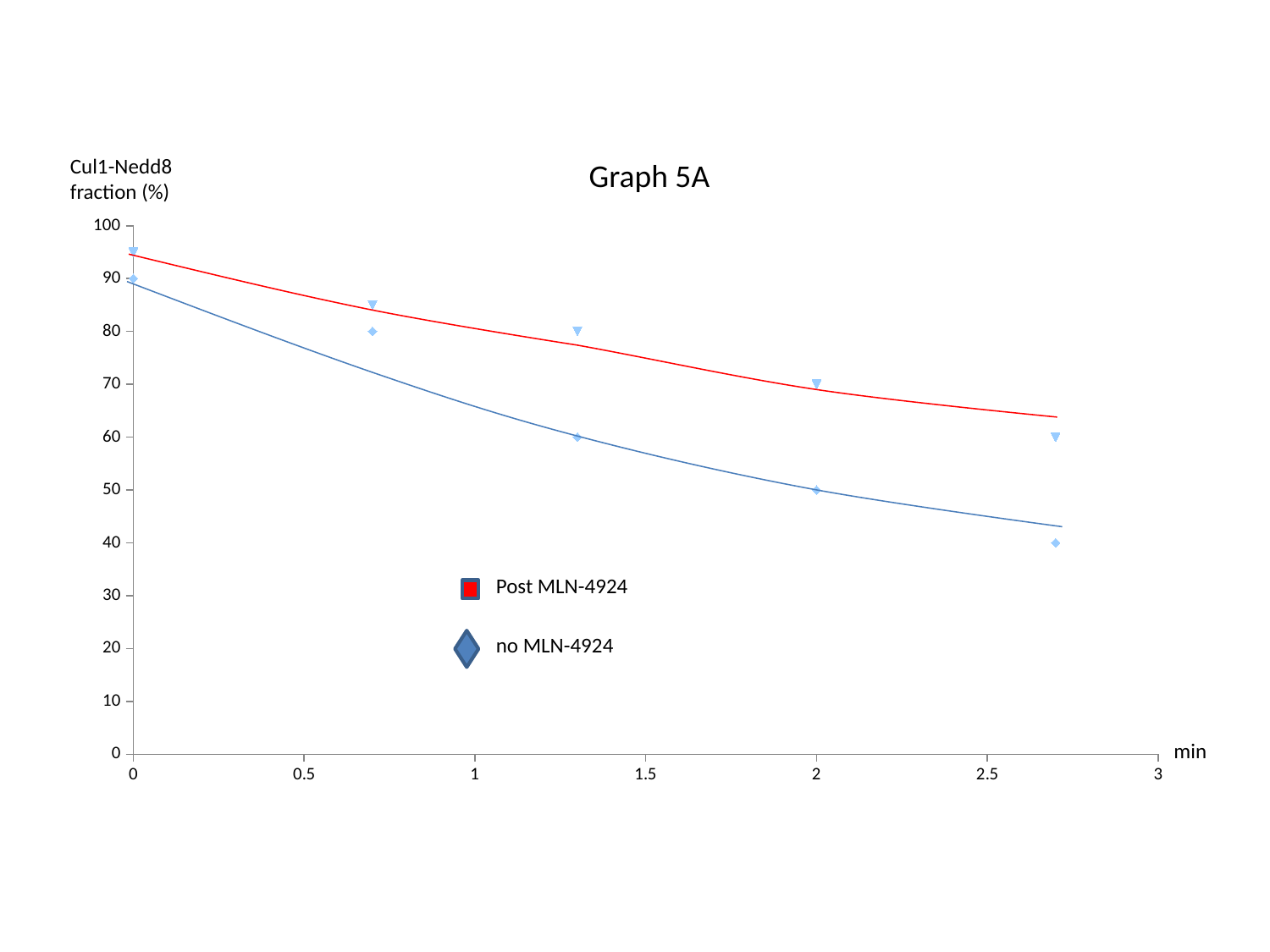

Cul1-Nedd8 fraction (%)
Graph 5A
### Chart
| Category | | |
|---|---|---|
Post MLN-4924
no MLN-4924
min

Supplement: Supplementary file 7 — Additional file 7. Graph 5A. [file 13008_2015_11_MOESM7_ESM.pptx]

## Slide 1
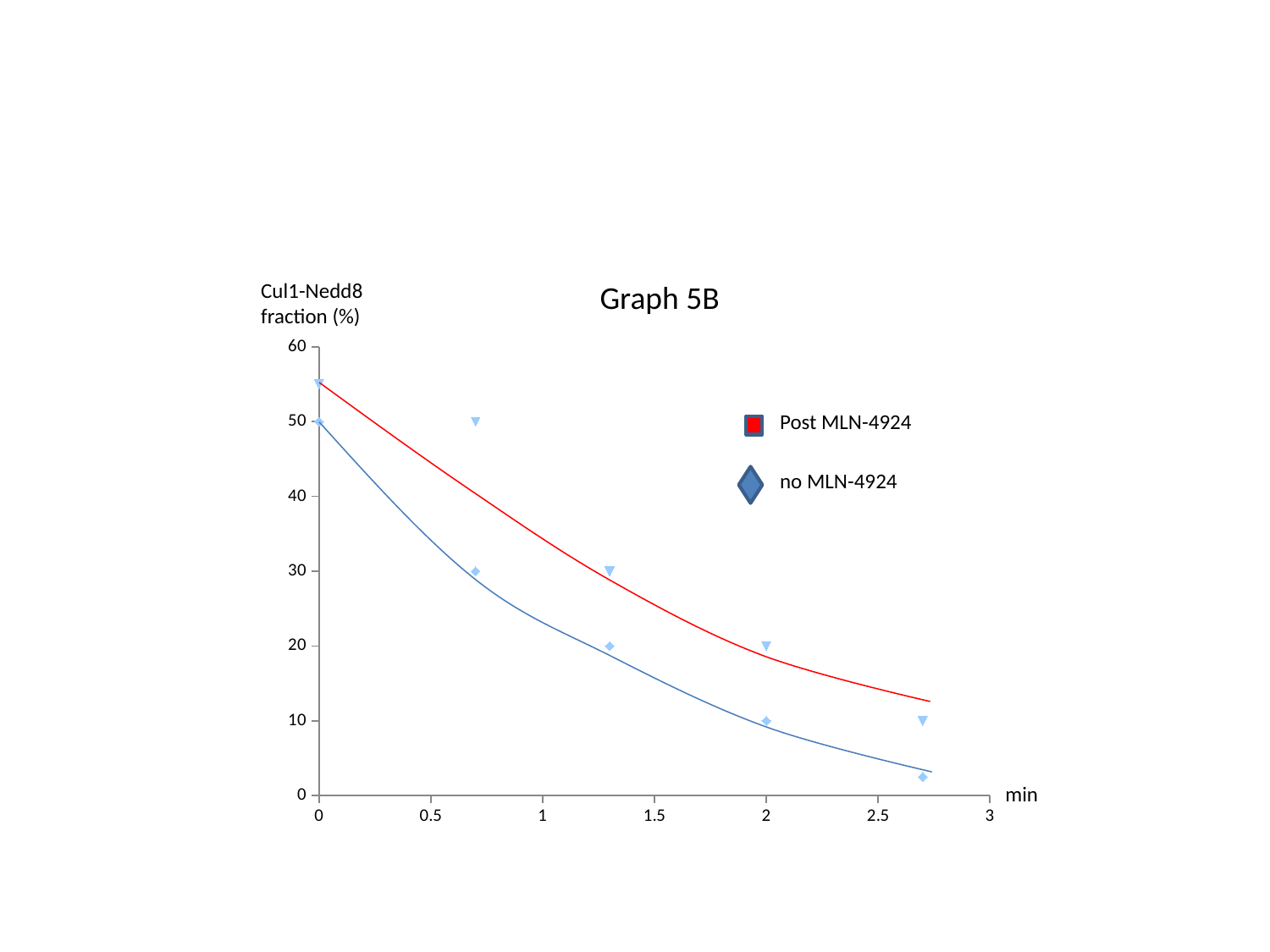

Cul1-Nedd8 fraction (%)
Graph 5B
### Chart
| Category | | |
|---|---|---|
Post MLN-4924
no MLN-4924
min

Supplement: Supplementary file 8 — Additional file 8. Graph 5B. [file 13008_2015_11_MOESM8_ESM.pptx]

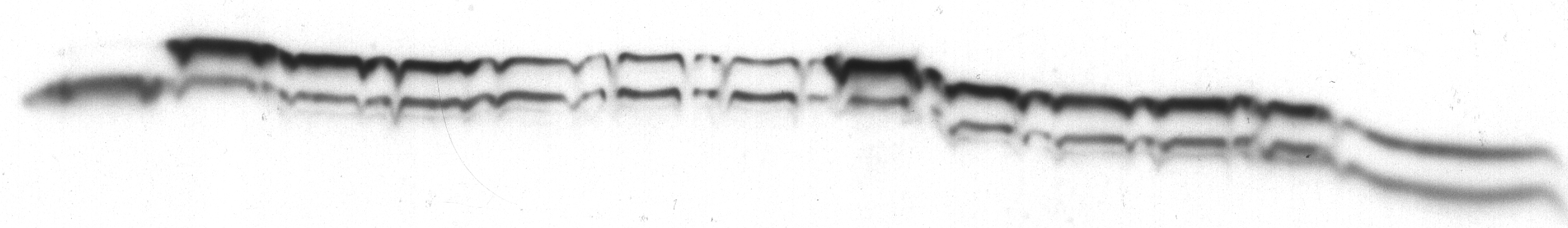

Supplement: Supplementary file 9 — Additional file 9. Uncropped Fig. 5a. [file 13008_2015_11_MOESM9_ESM.jpeg]

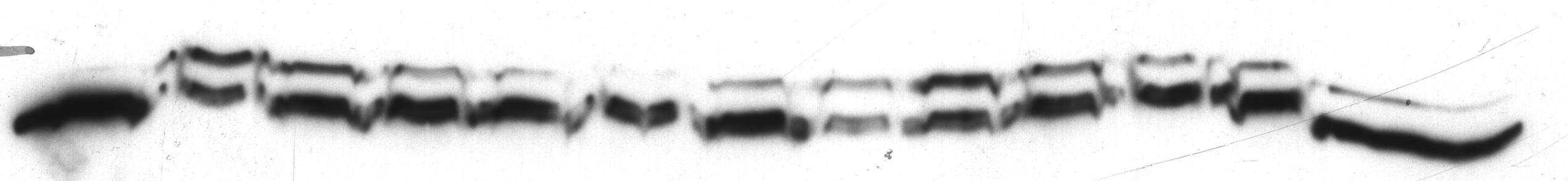

Supplement: Supplementary file 10 — Additional file 10. Uncropped Fig. 5b. [file 13008_2015_11_MOESM10_ESM.jpeg]
